# Bibliometric analysis of multimodal analgesia research in the perioperative period: trends, contributions, and emerging areas (2013–2023)

**DOI:** 10.3389/fmed.2025.1573112

**Published:** 2025-04-03

**Authors:** Wenchen Jiang, Yi Qin, Liang Chen

**Affiliations:** ^1^Department of Anesthesiology, Hunan Provincial Maternal and Child Health Care Hospital (Hunan Institute of Reproductive Medicine), Changsha, Hunan, China; ^2^Department of Anesthesiology, Xiangya Hospital, Central South University, Changsha, Hunan, China

**Keywords:** perioperative pain management, bibliometric analysis, CiteSpace, VOSviewer, multimodal analgesia

## Abstract

**Objectives:**

Multimodal analgesia (MA) is a cornerstone in perioperative pain management, enhancing pain relief and minimizing opioid consumption by targeting various pain pathways. This study conducts a bibliometric analysis of MA research from 2013 to 2023 to understand its development and impact on perioperative care.

**Methods:**

A comprehensive literature search of the Web of Science Core Collection (WOSCC) was conducted, covering publications from January 2013 to December 2023. Data were analyzed using VOSviewer and other bibliometric tools to identify publication trends, key contributors, and emerging research themes.

**Results:**

The analysis identified 1,939 studies on MA, with a notable increase in annual publications since 2020. The USA, China, and Canada were the leading contributors. Key terms like Non-Steroidal Anti-Inflammatory Drugs (NSAIDs), Enhanced Recovery After Surgery (ERAS), and Patient-Controlled Analgesia (PCA) were frequently associated with MA. Significant journals included the Cureus Journal of Medical Science and Anesthesia and Analgesia. Influential authors such as Richard D. Urman and Henrik Kehlet were highlighted for their contributions. The research showed significant advancements and growing global interest in MA.

**Conclusion:**

The study underscores the growing importance of MA in perioperative pain management, with significant contributions from leading countries and researchers. Future research should focus on optimizing pain management protocols, enhancing patient recovery, and reducing opioid dependency through MA.

## Introduction

Multimodal analgesia (MA) has become a cornerstone in perioperative pain management (PPM), recognized for its ability to enhance pain relief while minimizing opioid consumption (1–3). This approach utilizes a combination of analgesics targeting various pain pathways, providing a synergistic effect that improves pain control and reduces the adverse effects associated with high doses of single agents (4). This paradigm shift aligns with contemporary trends toward personalized and patient-centered care in medical practice.

Despite the established benefits of MA, there is a lack of comprehensive analysis regarding its current state in perioperative care, specifically the trends in research, key contributors, and emerging areas of focus. The rationale behind MA is rooted in the multifactorial nature of pain (5). By employing a mix of non-opioid analgesics (NOA) such as Non-Steroidal Anti-Inflammatory Drugs (NSAIDs), acetaminophen, local anesthetics (LA), and adjuvant drugs like gabapentinoids and N-Methyl-D-Aspartate receptor antagonists, healthcare providers can achieve more effective pain management (6). This strategy reduces reliance on opioids, thereby mitigating risks such as respiratory depression, nausea, constipation, and the potential for opioid dependence (7).

In the perioperative setting, effective pain management is essential not only for patient comfort but also for optimal recovery (8, 9). Inadequate pain control can lead to prolonged hospital stays, delayed mobilization, and an increased risk of developing chronic pain (10, 11). Consequently, MA protocols are integral to ERAS pathways, which emphasize early mobilization, shortened hospital stays, and improved overall outcomes (12, 13).

Bibliometrics, the statistical analysis of written publications, is vital for understanding scientific knowledge development (14). In medical research, it examines publication trends, citation patterns, and identifies key researchers and research areas. This method highlights important contributions and emerging themes, guiding future research and fostering collaboration. One of the key strengths of bibliometric analysis is its ability to systematically track the evolution of research in a specific field. In the context of MA, it offers valuable insights by mapping research trends over time, identifying influential authors, key publications, and emerging areas of interest. Additionally, bibliometrics can uncover gaps in the literature and provide guidance for future research in MA and its application in perioperative care.

Several bibliometric studies have explored pain management, including opioid-free pain strategies and the use of multimodal analgesia in various clinical settings. For example, Zin et al. (15) provided a bibliometric overview of opioid-related research trends, while Robert et al. (16) focused on the evolution of pain management protocols in surgical settings. However, these studies generally overlook the specific perioperative context of MA.

This bibliometric analysis aims to explore the landscape of multimodal analgesia in the perioperative period by identifying key research trends, influential authors, and major advancements in the field. Through mapping research contributions, this study seeks to offer insights into the current state of MA and provide direction for future research in perioperative pain management.

## Methods

### Search strategy

To ensure comprehensive coverage of relevant studies, a literature search was conducted in the Web of Science Core Collection (WOSCC) using the following search terms: TS = (“multimodal analgesia” OR “multimodal pain management” OR “combined analgesia”) AND TS = (“perioperative” OR “surgery” OR “surgical”). The search was limited to publications in English and covered articles and reviews published between January 1, 2013, and December 31, 2023. The search was performed on July 4, 2024, and the data were downloaded in TEXT format. The search was restricted to the Web of Science Core Collection (WOSCC) database to ensure the quality and relevance of the data used in the bibliometric analysis.

### Study selection

**Figure 1:**
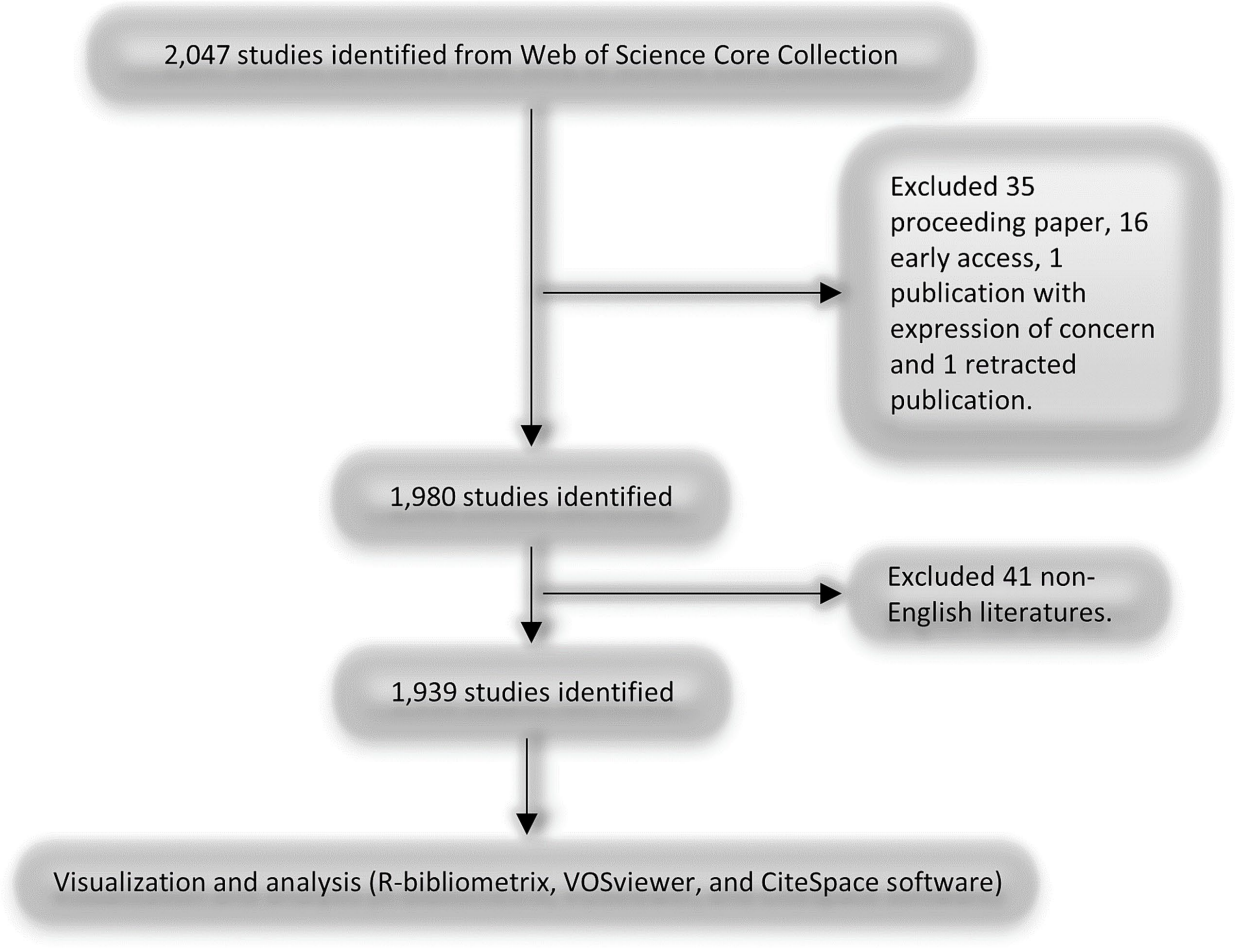
Flowchart of the search strategy and selection process for studies on multimodal analgesia in the perioperative period. This figure presents the step-by-step process of the literature search, screening, and selection criteria applied to identify relevant studies on multimodal analgesia during the perioperative period.

Studies were included if they were peer-reviewed original research articles or systematic reviews focused on multimodal analgesia in the perioperative period, specifically addressing pain management in surgical settings. Studies were excluded if they were case reports, case series, editorials, non-peer-reviewed publications, or published in languages other than English. The inclusion and exclusion criteria are shown in Figure 1.

### Data analysis

For our bibliometric analysis, we utilized the following software tools known for their robust methodologies:

1. VOSviewer (version 1.6.19): Developed by the Centre for Science and Technology Studies at Leiden University, VOSviewer is a free tool for constructing and visualizing bibliometric networks (17). We applied VOSviewer to construct collaboration networks, co-citation analyses, and keyword co-occurrence networks, which helped us identify emerging research themes and key patterns in multimodal analgesia during the perioperative period (18–20).
2. CiteSpace (version 6.2.R4): Created by Dr. Chaomei Chen at Drexel University, CiteSpace is a Java application available under a freeware license (21). We specifically used CiteSpace to detect citation bursts and to analyze the structure of the research field through cluster analysis and dual-map overlays. This allowed us to map out significant research milestones and key developments in the field (22, 23). CiteSpace uses several structural metrics, such as betweenness centrality, modularity, and silhouette scores, to analyze co-citation networks. Betweenness centrality measures a node’s role as a bridge in the network, with higher values indicating influential literature. Modularity assesses the division of a network into distinct communities, with values above 0.3 indicating significant division. The silhouette score evaluates the coherence of nodes within clusters, with values above 0.5 indicating credible cohesion (24).
3. Bibliometrix R package (version 3.2.1): This open-source R package is designed for comprehensive bibliometric analysis. It supports various quantitative research activities, including data processing, descriptive analysis, and network analysis. The tool generates essential metrics such as citation counts, collaboration indices, and impact measures, helping to develop a detailed understanding of research dynamics. Additionally, Bibliometrix assesses journal impact factors based on the latest Journal Citation Reports (JCR) (25).

These tools were selected for their ability to handle extensive bibliographic data and their specific analytical strengths, which facilitate a deeper understanding of the patterns, trends, and structural relationships within the field of MA during the perioperative period.

## Results

### Publication trends in MA research

**Figure 2:**
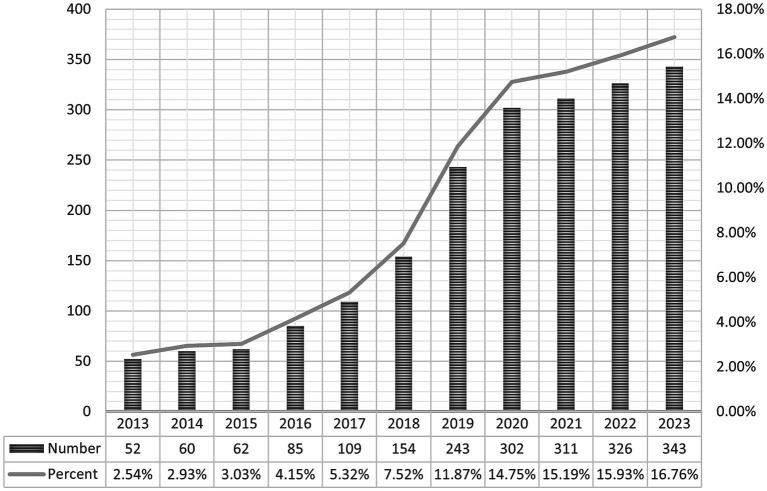
Publication trends in multimodal analgesia research during the perioperative period. The figure illustrates the annual growth in the number of publications related to multimodal analgesia in the perioperative setting, highlighting the increasing interest and research activity in this area over time.

From 2013 to 2023, our bibliometric analysis identified 1,939 studies on MA in the perioperative period, comprising 1,526 articles and 413 reviews. Annual publications have shown an upward trend, with a significant surge since 2020, when they surpassed 300 per year, reflecting the growing prominence of this field (Figure 2).

### Geographical and institutional distribution

**Figure 3:**
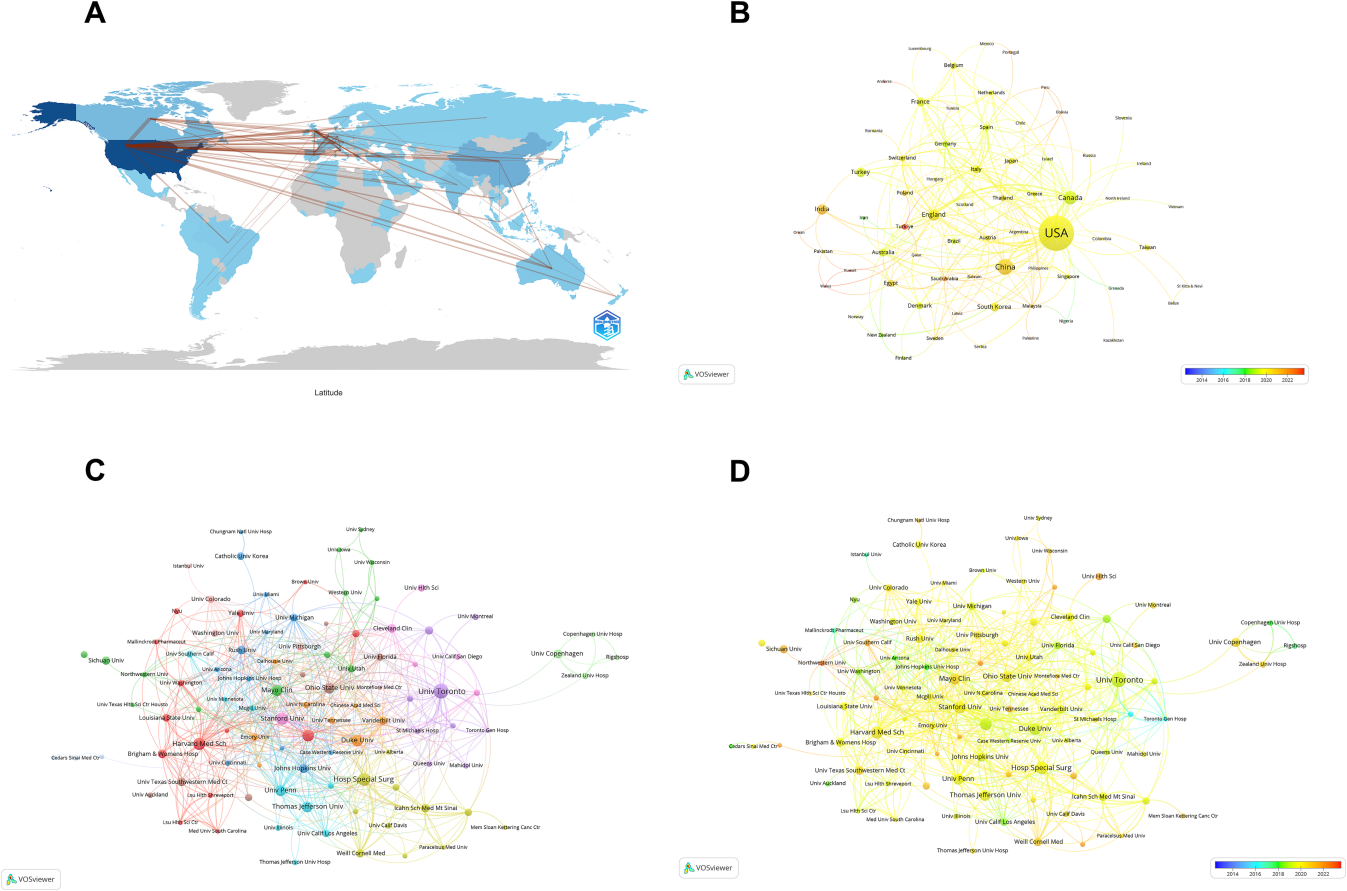
Geographical collaboration and publication analysis. **(A)** A global map depicting the collaboration networks between countries in multimodal analgesia research. **(B)** An analysis of the number of publications by country over recent years, showing leading contributors. **(C)** A visual representation of collaborating institutions on a global scale. **(D)** An analysis of institutional publication outputs over recent years, emphasizing key research centers in the field.

The research spans 84 countries and 2,471 institutions. The USA, China, and Canada are the largest contributors, with 40.25, 8.07, and 5.94% of total publications, respectively. These countries contribute over half of the total publications, underscoring significant interest in perioperative MA. Network analysis (Figure 3A) indicates robust collaboration, particularly between the USA, China, Canada, and England. The USA holds a more influential position in the research network with a centrality index of 0.55, compared to China’s 0.03. Temporal trends (Figure 3B) reveal a shift in research epicenter, with China and India increasing publication volumes in recent years, following earlier dominance by the USA and Canada (Table 1).

**Table 1:** The top 10 countries/regions on research of MA in the perioperative period.

Rank	Country	Publications	Proportion of publications (%)	Centrality
1	USA	908	40.25	0.55
2	China	182	8.07	0.03
3	Canada	134	5.94	0.08
4	England	91	4.03	0.20
5	India	78	3.46	0.03
6	Turkey	65	2.88	0.00
7	France	56	2.48	0.08
8	South Korea	54	2.39	0.00
9	Italy	46	2.04	0.10
10	Australia	45	1.99	0.05

Institutional contributions highlight the dominance of US institutions, with the University of California System leading in document count (5.67%). The University System of Ohio and the University of Toronto, with centrality indices of 0.11 and 0.10, respectively, play significant integrative roles within the research community (Table 2). A collaborative network of 116 institutions, based on a publication threshold of six, shows close cooperation among select universities (Figures 3C,D).

**Table 2:** The top 10 institutions on research of MA in the perioperative period.

Rank	Institution	Belong Country	Publications	Proportion of publications (%)	Centrality
1	University of California System	USA	110	5.67	0.08
2	Harvard University	USA	109	5.62	0.06
3	University of Texas System	USA	61	3.15	0.07
4	University of Toronto	Canada	58	2.99	0.10
5	University System of Ohio	USA	48	2.48	0.11
6	Stanford University	USA	41	2.11	0.02
7	Duke University	USA	38	1.96	0.08
8	Cornell University	USA	36	1.86	0.02
9	Johns Hopkins University	USA	35	1.81	0.09
10	Jefferson University	USA	35	1.81	0.02

### Journals and co-cited journals

**Figure 4:**
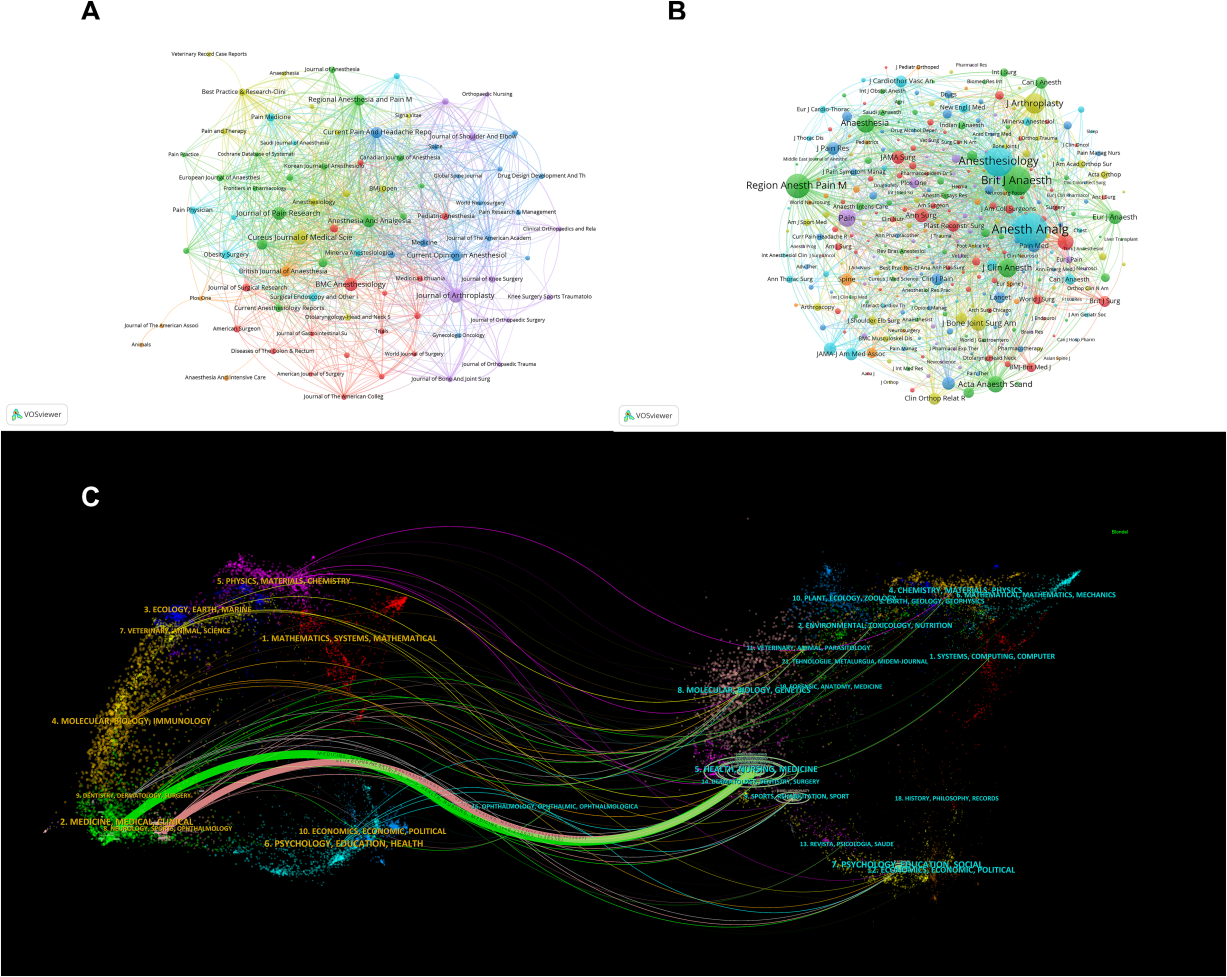
Journal contribution and citation analysis in multimodal analgesia research. **(A)** Visualization of journals that contribute to research on multimodal analgesia. **(B)** A map of co-cited journals, reflecting inter-journal citation patterns. **(C)** Dual-map overlay showing the relationship between citing and cited journals, providing insights into the interdisciplinary nature of the research.

Research concentration in specific scientific journals is apparent (Table 3; Figure 4A). The Cureus Journal of Medical Science leads with 54 publications, followed by the Journal of Arthroplasty and BMC Anesthesiology. These journals have impact factors (IFs) ranging from 1 to 9.1 and are primarily in the first and second quartiles of the JCR 2023, indicating their influence. Co-citation analysis shows Anesthesia and Analgesia as the most cited journal (4,505 citations), followed by Anesthesiology (3,373 citations) and the British Journal of Anaesthesia (3,133 citations), highlighting their pivotal roles in MA research (Figure 4B; Table 4).

**Table 3:** The top 10 productive journals related to MA in the perioperative period.

Rank	Journal	Publications	IF (JCR2023)	JCR quartile
1	Cureus Journal of Medical Science	54	1	Q3
2	Journal of Arthroplasty	52	3.4	Q1
3	BMC Anesthesiology	42	2.3	Q2
4	Journal of Pain Research	42	2.5	Q2
5	Anesthesia and Analgesia	39	4.6	Q1
6	Current Opinion in Anesthesiology	32	2.3	Q2
7	Regional Anesthesia and Pain Medicine	32	5.1	Q1
8	Current Pain and Headache Reports	31	3.2	Q2
9	Journal of Clinical Anesthesia	30	5	Q1
10	British Journal of Anaesthesia	27	9.1	Q1

**Table 4:** Top 10 journals for co-citation of MA in the perioperative period.

Rank	Cited journal	Citation	IF (JCR2022)	JCR quartile
1	Anesthesia and Analgesia	4,505	4.6	Q1
2	Anesthesiology	3,373	9.1	Q1
3	British Journal of Anaesthesia	3,133	9.1	Q1
4	Regional Anesthesia and Pain Medicine	2,267	5.1	Q1
5	Journal of Arthroplasty	1,606	3.4	Q1
6	Journal of Clinical Anesthesia	1,332	5	Q1
7	Anaesthesia	1,248	7.5	Q1
8	Pain	1,187	5.9	Q1
9	ACTA Anaesthesiologica Scandinavica	1,031	1.9	Q2
10	Cochrane Database of Systematic Reviews	987	8.8	Q1

The dual-map overlay of journals in Figure 4C demonstrates the relationship between citing and cited journals. The most prominent paths indicate that journals in the Health/Nursing/Medicine domain are frequently cited by publications in the Medicine/Medical/Clinical and Neurology/Sports/Ophthalmology domains, effectively representing the interconnectedness between citing and cited journals across various fields.

### Authors and co-cited authors

**Figure 5:**
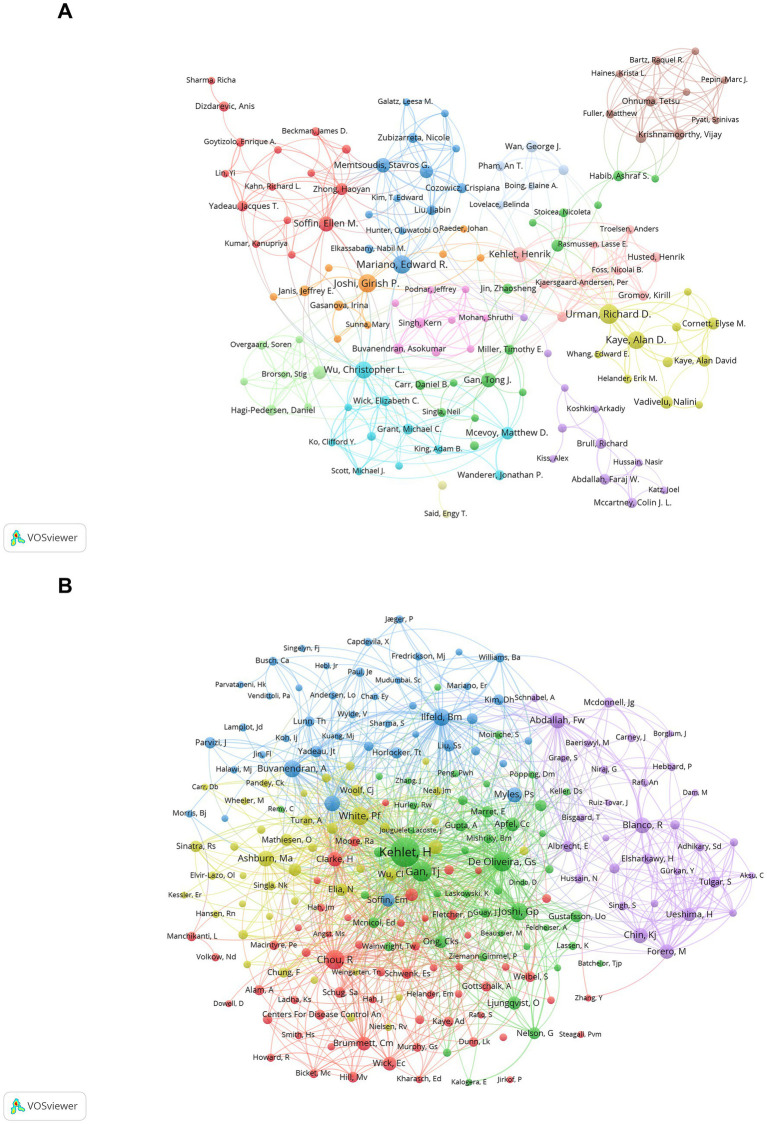
Analysis of authors and co-cited authors in multimodal analgesia research. **(A)** Visualization of the most prolific authors contributing to perioperative multimodal analgesia research. **(B)** A map of co-cited authors, illustrating the key contributors whose work has had significant influence in the field.

Our bibliometric analysis mapped the research landscape on MA in the perioperative period, identifying both prolific authors and frequently co-cited ones (Figures 5A,B). Table 5 highlights the top 10 authors by publication count, with Richard D. Urman leading at 18 publications. Notably, seven of these top authors are based in the USA, highlighting the significant contributions from this country. Co-citation data reveals Henrik Kehlet as the most influential author, with 541 citations, emphasizing his substantial impact in this field.

**Table 5:** Top 10 authors and co-cited authors on research of MA in the perioperative period.

Rank	Authors	Location	Publications	Co-cited authors	Location	Citations
1	Urman, Richard D.	USA	18	Kehlet, Henrik	Denmark	541
2	Joshi, Girish P.	USA	16	Gan, Tong J.	Canada	258
3	Mariano, Edward R.	USA	16	Chou, R.	USA	241
4	Kaye, Alan D.	USA	15	de Oliveira, Gabriel S.	Brazil	237
5	Wu, Christopher L.	USA	14	White, Paul F.	USA	206
6	Kehlet, Henrik	Denmark	11	Joshi, Girish P.	USA	202
7	Soffin, Ellen M.	USA	11	Ilfeld, Brian M.	USA	187
8	Gan, Tong J.	Canada	10	Buvanendran, Asokumar	USA	185
9	Memtsoudis, Stavros G.	USA	10	Blanco, Ricardo	Spain	164
10	Mathiesen, Ole	Denmark	9	Abdallah, Faraj W.	Canada	157

### Co-cited references and reference with citation bursts

**Figure 6:**
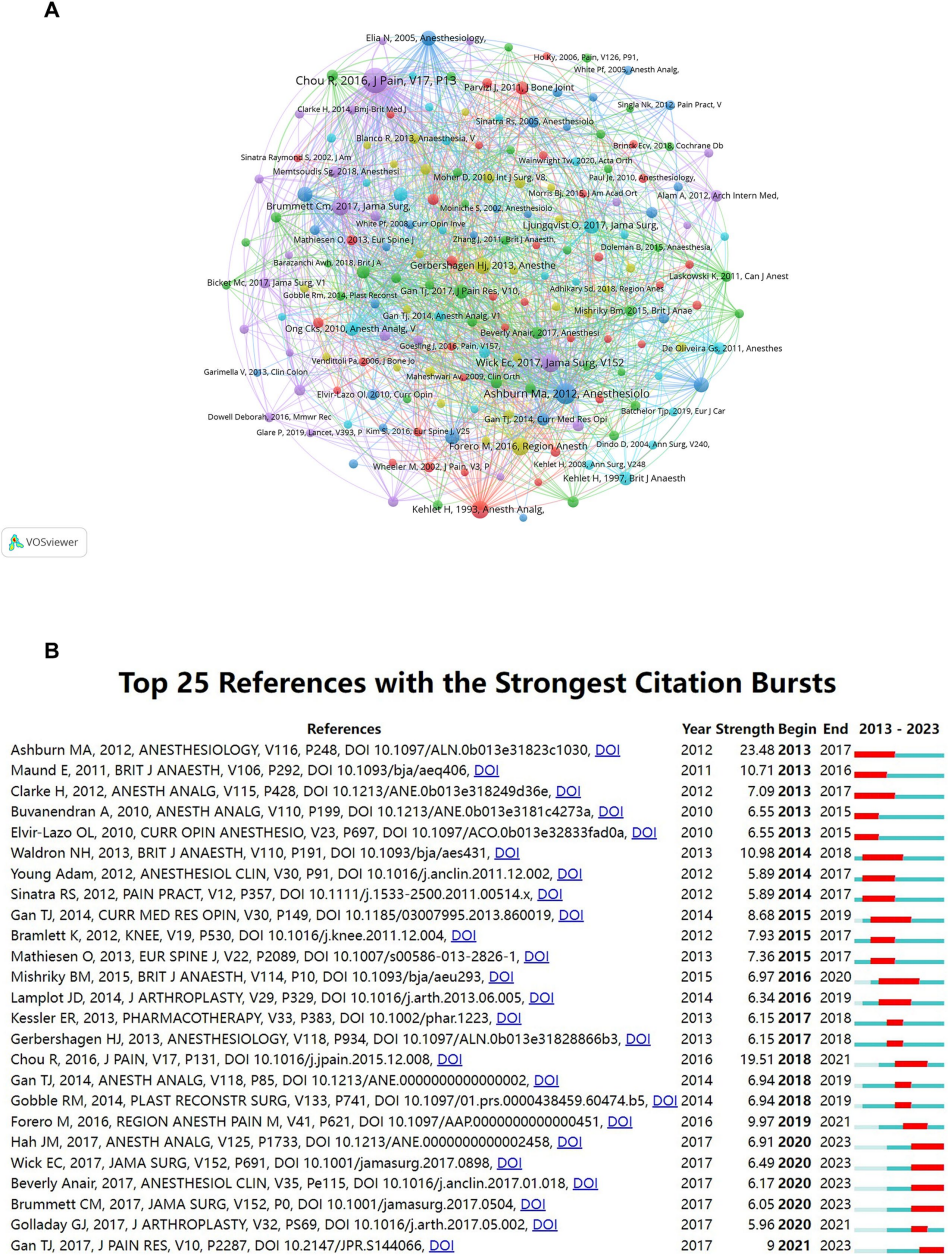
Reference citation analysis in multimodal analgesia research. **(A)** Visualization of the most frequently co-cited references in the field. **(B)** A timeline showcasing the top 25 references with the most significant citation bursts, indicating highly influential works over time.

Our study pinpointed the most influential works in MA during the perioperative period based on co-citation frequencies. Table 6 lists the top 10 co-cited references, led by Roger Chou’s 2016 Journal of Pain article, “Management of Postoperative Pain: A Clinical Practice Guideline,” with 223 citations. This foundational work has significantly shaped subsequent research in the field. These leading articles, many published in prestigious journals such as Lancet and JAMA Surgery, account for four of the top 10 co-cited references (Table 6; Figure 6A).

**Table 6:** Ranking of the top 10 co-cited references for MA in the perioperative period.

Rank	Reference	Citation	Year	First Author	Journal
1	Management of Postoperative Pain: A Clinical Practice Guideline From the American Pain Society, the American Society of Regional Anesthesia and Pain Medicine, and the American Society of Anesthesiologists’ Committee on Regional Anesthesia, Executive Committee, and Administrative Council	223	2016	Chou, Roger	Journal of Pain
2	Practice Guidelines for Acute Pain Management in the Perioperative Setting *An Updated Report by the American Society of Anesthesiologists Task Force on Acute Pain Management*	146	2012	Ashburn, Michael A.	Anesthesiology
3	Postoperative Multimodal Analgesia Pain Management With Nonopioid Analgesics and Techniques A Review	117	2017	Wick, Elizabeth C.	JAMA Surgery
4	The Erector Spinae Plane Block *A Novel Analgesic Technique in Thoracic Neuropathic Pain*	104	2016	Forero, Mauricio	Regional Anesthesia and Pain Medicine
5	The value of “multimodal” or “balanced analgesia” in postoperative pain treatment.	104	1993	Kehlet, Henrik	Anesthesia and Analgesia
6	New Persistent Opioid Use After Minor and Major Surgical Procedures in US Adults	99	2017	Brummett, Chad M.	JAMA Surgery
7	Pain Intensity on the First Day after Surgery *A Prospective Cohort Study Comparing 179 Surgical Procedures*	92	2013	Gerbershagen, Hans J.	Anesthesiology
8	Enhanced Recovery After Surgery: A Review	85	2017	Ljungqvist, Olle	JAMA Surgery
9	Does multimodal analgesia with acetaminophen, nonsteroidal antiinflammatory drugs, or selective cyclooxygenase-2 inhibitors and patient-controlled analgesia morphine offer advantages over morphine alone?: Meta-analyses of randomized trials	82	2005	Elia, N	Anesthesiology
10	Persistent postsurgical pain: risk factors and prevention	82	2006	Kehlet, Henrik	Lancet

The “citation burst” analysis identified articles that experienced a rapid increase in citations over specific periods, indicating the emergence of significant research topics (26). Our analysis highlighted the top 25 references with notable citation bursts (Figure 6B), signaling growing interest from 2013 to 2023. Michael A. Ashburn’s paper, “Practice guidelines for acute pain management in the perioperative setting,” showed the strongest citation burst (strength = 23.48) from 2013 to 2017. This comprehensive guideline offers evidence-based recommendations for acute pain management during the perioperative period. Its widespread adoption has significantly influenced pain management protocols. Roger Chou et al.’s work, “Management of Postoperative Pain,” ranked second (strength = 19.51) from 2018 to 2021. This multidisciplinary guideline provides recommendations for postoperative pain management, emphasizing multimodal approaches. Its citation increase reflects its role in standardizing postoperative pain care. N.H. Waldron’s paper, “Impact of Perioperative Dexamethasone on Postoperative Analgesia and Side-Effects: Systematic Review and Meta-Analysis,” demonstrated a significant citation burst (strength = 10.98) from 2014 to 2018. This meta-analysis evaluates the efficacy of dexamethasone in enhancing postoperative analgesia and reducing side effects. The surge in citations highlights its substantial contribution to refining postoperative care strategies. The significant rise in citations of these articles underscores their pivotal role in advancing MA research. They have not only influenced clinical practices and informed guidelines but have also spurred further research in the field. Analyzing such articles provides insight into key developments and shifts in research focus within MA.

### Hotspots and Frontiers

**Figure 7:**
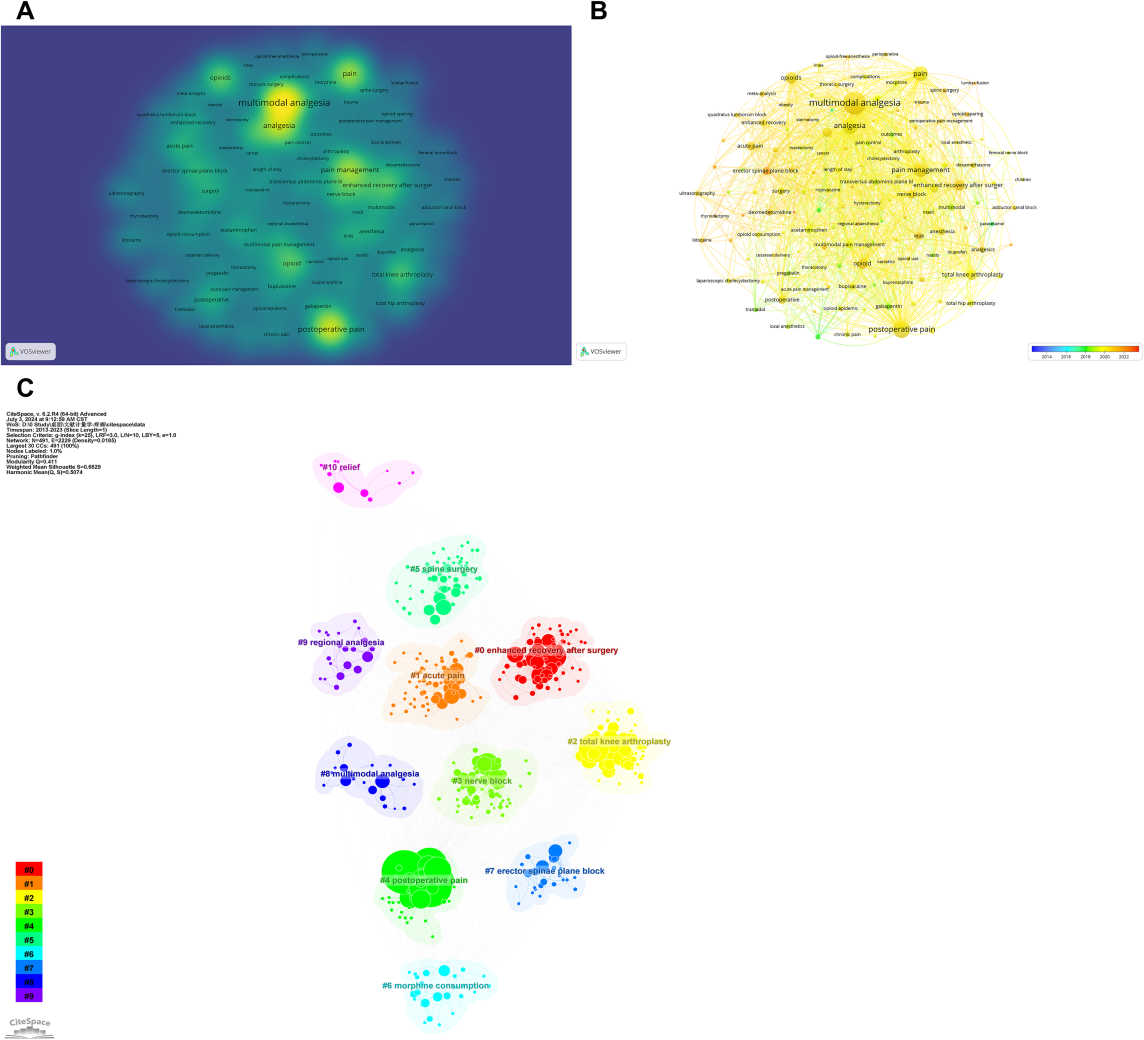
Keyword analysis in multimodal analgesia research. **(A)** Visualization of the most frequently used keywords in multimodal analgesia studies. **(B)** A temporal map showing the evolution of keyword usage over time, reflecting trends and shifts in research focus. **(C)** Clustering of related keywords, revealing key thematic areas and research topics within the field.

Using VOSviewer and CiteSpace, we analyzed 2,773 author keywords across 1,939 documents, identifying 134 keywords appearing in at least nine documents each. Our analysis, which included keyword co-occurrence and temporal progression mapping, revealed evolving trends in MA during the perioperative period. “MA “emerged as a central node in the co-occurrence network, frequently cited in the literature. Significant adjacent terms included “opioids,” “total knee arthroplasty (TKA),” “sternotomy,” and “ERAS,” indicating their importance in the field (Figure 7A). The temporal progression map highlighted emerging research areas, with terms like “erector spinae plane block,” “dexmedetomidine,” and “thyroidectomy” appearing in orange, marking them as frontier topics (Figure 7B). In contrast, foundational terms such as “paracetamol” and “tramadol” were depicted in green, providing historical context to these evolving trends. CiteSpace cluster analysis identified 11 clusters, ranging from “#0 ERAS “to “#10 relief,” with a modularity (Q) value above 0.3 and a silhouette (S) value above 0.5 (Q = 0.51, S = 0.66), confirming the robustness and significance of these clusters (Figure 7C).

## Discussion

### General information

The annual publication trend, as illustrated in Figure 2, highlights a growing academic interest in this field. Since 2016, there has been a consistent rise in publications, with the last 5 years accounting for 74.5% of the total output, signifying rapid advancement. This trend can be attributed to several key factors. In particular, the opioid crisis in the United States has led to significant policy changes, such as the implementation of the 2016 “Opioid Prescribing Guidelines” by the Centers for Disease Control and Prevention (CDC) (27), which has driven research into opioid alternatives and MA. Additionally, the introduction of the “Comprehensive Addiction and Recovery Act” (CARA) (28) in 2016, which promotes non-opioid pain management strategies, further stimulated research in this area. The increasing adoption of ERAS protocols, which emphasize opioid-sparing strategies including MA, has also contributed to the surge in publications related to perioperative pain management.

The United States stands out as the most prolific country, with five of the 10 most cited authors, establishing its leading position in this domain. Anesthesia and Analgesia emerges as the most cited journal and the fifth most published, serving as a significant reference for scholars.

The chord diagram reveals that the United States, the United Kingdom, and Canada have the widest chords, indicating their strong collaborative ties with a diverse range of countries (Supplementary Figure 1). This observation is further supported by the analysis of the top 10 co-cited references, half of which were authored by researchers affiliated with these nations. This substantial overlap underscores a significant correlation between the intensity of international collaboration and the quality of research. The close ties among these countries suggest that enhanced cooperation not only facilitates the exchange of knowledge and resources but also contributes to the production of high-impact research, reinforcing the notion that collaboration is a key driver of academic excellence.

As depicted in Figure 5B and Table 5, five of the top 10 co-cited authors and nine of the top 10 authors hail from the United States. Notably, Henrik Kehlet of Copenhagen University, the most co-cited author, led the seminal work “The Value of ‘Multimodal’ or ‘Balanced Analgesia’ in Postoperative Pain Treatment” (29). Tong J. Gan of Stony Brook University Renaissance School of Medicine is renowned for his pivotal contributions to perioperative MA through research, education, and clinical practice, significantly enhancing postoperative pain management and patient recovery experiences (30–32). Roger Chou of Oregon Health & Science University, as the lead author of the influential article “Management of Postoperative Pain: A Clinical Practice Guideline” from the American Pain Society and other organizations, has extensively contributed to perioperative MA through research, systematic reviews, and guidelines that have shaped clinical practice and improved patient outcomes (1, 33, 34).

Table 5 reveals that top co-cited references predominantly focus on perioperative and postoperative pain management, particularly MA strategies. These references explore combining various NOA and techniques to enhance pain control, reduce opioid usage and its side effects, and improve patient recovery outcomes.

### Surgical applications

In the first cluster, ‘Surgical Applications’, the emphasis is placed on specific surgical procedures where MA has demonstrated a significant impact. Noteworthy procedures include TKA, Spine Surgery, and protocols under ERAS. TKA, a prevalent orthopedic surgery, involves the replacement of the knee joint with prosthetic components (35). The complexity of TKA necessitates effective pain management to facilitate early mobilization and rehabilitation, which are pivotal for favorable patient outcomes. MA, incorporating a variety of analgesic methods and medications, has been effective in mitigating postoperative pain and enhancing functional recovery in TKA patients (1, 36). Over time, research has evolved to emphasize opioid-sparing strategies in TKA, particularly with the integration of nerve blocks and regional anesthesia techniques, reducing dependency on systemic analgesics (37).

Similarly, spine surgery has gained prominence in pain management discussions due to the increasing complexity of surgical interventions and their associated postoperative pain challenges. The range from minimally invasive techniques to extensive spinal fusions, all of which can potentially cause significant postoperative pain (38, 39). Effective pain management is crucial for facilitating early rehabilitation, reducing the risk of developing chronic pain, and improving patient satisfaction (40, 41). MA in spine surgery typically involves the use of LA, systemic analgesics (SA), and regional techniques such as epidural analgesia (42). Recent literature has highlighted the role of multimodal approaches in spine surgery, shifting from traditional opioid-centric regimens to strategies incorporating non-opioid analgesics and regional blocks, reflecting a broader trend toward personalized pain control. One study examined the efficacy of a combination of NOA (acetaminophen and gabapentin) and intraoperative infusions (ketamine and lidocaine), finding that it did not significantly improve recovery or reduce opioid consumption in patients undergoing multilevel spine surgery (43). Furthermore, advancements in regional anesthesia techniques, such as ultrasound-guided methods, have been shown to decrease the risks associated with local anesthetic systemic toxicity and nerve injury (44). These findings highlight the importance of precision in regional anesthesia and underscore the necessity of ongoing research to determine the most effective strategies for reducing opioid-related adverse effects in spine surgery.

ERAS protocols offer a holistic framework for perioperative care, designed to enhance the surgical experience through a blend of evidence-based interventions (45, 46). These protocols aim to reduce the physiological impact of surgery, hasten recovery, and improve overall outcomes by integrating comprehensive care steps that span patient education, nutrition optimization, precise analgesic and anesthetic plans, and early mobilization (46). The incorporation of MA into ERAS protocols has gained traction as a central component of pain management, demonstrating a paradigm shift in perioperative care. A key pillar of ERAS is the implementation of MA, which is central to effective pain management strategies (47). Effective implementation of MA under ERAS protocols significantly enhances patient comfort, facilitates a faster return to function, and allows for earlier engagement in physical therapy, reducing discomfort and expediting recovery (48). This active participation is crucial in preventing post-surgical complications such as deep vein thrombosis (DVT), which are common in patients with prolonged immobility (49). In the framework of ERAS, the tailored approach to MA is continuously refined based on ongoing research and feedback from clinical practice. This adaptive methodology ensures that each patient’s pain management regimen is optimized to their specific surgical procedure and individual needs, promoting faster healing and reducing the overall burden of surgery (50, 51). The increasing emphasis on ERAS in recent years signifies a transition toward structured, protocol-driven perioperative care, where MA plays a pivotal role in ensuring enhanced recovery and opioid stewardship.

### Pain management techniques and strategies

In the second cluster, “Pain Management Techniques and Strategies,” the focus shifts to various methods that enhance PPM through MA. Key techniques within this cluster include Nerve Block Techniques, Quadratus Lumborum Block, Regional Analgesia, and MA, reflecting a shift from systemic opioid reliance to targeted, site-specific pain control approaches.

Nerve block techniques are integral to the MA paradigm, offering targeted analgesia that significantly enhances PPM (52). These techniques involve the precise administration of anesthetics adjacent to specific nerves, thereby attenuating pain signals at their origin. The use of brachial plexus blocks in upper extremity surgeries exemplifies their efficacy, reducing reliance on systemic opioids and significantly improving postoperative analgesia (53), reflects an increasing emphasis on precision pain control and opioid minimization. In the perioperative context, brachial plexus blocks facilitate quicker recovery by enabling effective pain control, which is critical for patient mobilization and rehabilitation (54). Combining femoral and sciatic nerve blocks, commonly employed in lower extremity surgeries, supports early mobilization and rehabilitation by providing superior pain control compared to other methods, thus playing a crucial role in perioperative care strategies, though more research is needed on rehabilitation parameters (55). Thoracic interfascial block is effective for alleviating thoracic cage pain in intensive care unit patients, contributing to enhanced functional recovery post-surgery (56). These nerve blocks exemplify targeted interventions that align with the objectives of reducing opioid use and expediting patient recovery, critical components of modern perioperative care. The Erector Spinae Plane Block (ESPB) is used in thoracic, abdominal, and hip surgeries to mitigate visceral and somatic pain by anesthetizing the abdominal wall (57, 58). This regional technique significantly lowers postoperative pain scores and decreases opioid requirements, facilitating enhanced recovery protocols (59). The inclusion of these techniques in recent research suggests a paradigm shift toward less invasive yet highly effective regional anesthesia methods, driven by technological advancements and clinical demand.

Regional analgesia encompasses a range of techniques that provide extensive pain relief by targeting central and peripheral nerve pathways directly associated with the surgical site (60). Epidural analgesia and spinal anesthesia are pivotal in managing pain for lower extremity and lower abdominal surgeries, offering profound pain relief and reducing the surgical stress response (61). In the perioperative phase, these techniques are essential for optimizing patient comfort and minimizing stress-related complications. Continuous peripheral nerve blocks provide sustained analgesia, crucial for managing pain in procedures such as orthopedic surgeries or surgeries involving major joints (62). Their integration into perioperative care protocols aligns with the goals of MA to improve patient outcomes and satisfaction, ensuring a holistic approach to pain management.

Over the years, the conceptualization of MA has evolved from a simple multimodal approach to a highly individualized, evidence-driven strategy aimed at optimizing recovery and minimizing opioid-related complications. MA approach involves using multiple analgesic medications and techniques targeting different pain pathways to maximize pain relief while minimizing reliance on any single type of medication, particularly opioids. Combining various analgesics, such as acetaminophen, NSAIDs, and selective COX-2 inhibitors, with patient-controlled analgesia (PCA) morphine, has been shown through meta-analyses to offer superior pain relief and reduce opioid-related side effects compared to morphine alone (1). Advocated by guidelines from the American Pain Society and related organizations (1), this strategy aligns with the principles of ERAS, aiming to expedite recovery and improve overall patient outcomes. By addressing different aspects of pain through pharmacological and non-pharmacological methods, MA leads to more effective and balanced pain control, contributing significantly to the overarching goals of perioperative care and facilitating faster patient recovery and improved surgical outcomes (63, 64).

### Pain and medication consumption

In the third cluster, “Pain and Medication Consumption,” the focus is on managing perioperative pain and optimizing medication use through MA. This section includes key areas such as Acute Pain, Postoperative Pain, and Morphine Consumption.

Acute pain, occurring immediately after surgery, significantly impacts patient recovery and satisfaction (65–67). As clinical guidelines continue to evolve, there has been a notable transition from opioid-centric acute pain management to multimodal and patient-specific strategies, incorporating regional analgesia, non-opioid analgesics, and integrative pain management techniques (48). Effective management of acute pain is essential to prevent the development of chronic pain and to promote early mobilization and rehabilitation (68). MA plays a crucial role in this context, as it provides balanced and comprehensive pain relief by combining different analgesic agents and techniques. This approach enhances patient comfort, facilitates faster recovery, and reduces postoperative complications by addressing pain through various mechanisms.

Postoperative pain management is critical for recovery, mobility, and overall patient outcomes (40, 69). Uncontrolled postoperative pain can lead to delayed recovery, increased risk of complications, and prolonged hospital stays (10, 11). MA combines various analgesic techniques and medications to offer effective pain control, reduce opioid consumption, and improve patient satisfaction (6). Strategies for managing postoperative pain include the use of LA, regional analgesia techniques, SA, and non-pharmacologic interventions such as physical therapy and cognitive-behavioral therapy (7). These comprehensive pain management approaches promote better functional recovery and overall patient outcomes.

Reducing morphine consumption is a central focus within the context of MA, given the risks associated with opioid use, such as respiratory depression, nausea, vomiting, and the potential for addiction (6, 7). Studies have demonstrated that MA protocols can significantly reduce opioid consumption, decrease the incidence of opioid-related side effects, and improve overall patient outcomes (70, 71). A growing body of literature also supports the role of patient education and shared decision-making in optimizing perioperative pain control while minimizing unnecessary opioid exposure. By optimizing pain control while reducing opioid use, MA promotes safer and more effective perioperative care.

In this study, we conducted a bibliometric analysis of MA research published between 2013 and 2023. Our findings reveal both consistencies and deviations when compared to previous studies in the field. Our focus on the 2013–2023 period aligns with certain studies emphasizing pain management across various medical contexts. For instance, a bibliometric analysis covering a broader timeframe identified significant growth in research, with recent trends focusing on e-health, telemedicine, virtual reality, and peripheral nerve blocks (72). However, by concentrating on a specific decade, our study offers a more nuanced understanding of the evolution of MA, enabling detailed observation of trends and characteristics during this period. Our findings indicate that the United States, China, and Canada are the primary contributors to MA research. This aligns with the dominant role of developed countries in medical research, as highlighted in previous studies. For example, in bibliometric analyses of labor analgesia, the United States is also one of the countries with the highest number of publications (73). However, our study further underscores the significant role of China in MA research, likely reflecting the country’s increasing emphasis and investment in pain management in recent years. Compared to bibliometric analyses in other fields, the contributions of different countries to MA research may vary due to differences in medical resources, research priorities, and policy support. The frequent occurrence of terms such as NSAIDs, ERAS, and PCA in our analysis aligns with their established importance in pain management literature (74). For example, multimodal analgesia protocols have been associated with improved outcomes in various surgical procedures, including total knee arthroplasty (75). Moreover, advances in molecular mechanisms have led to the development of new pharmaceutical products to treat postoperative pain, highlighting the ongoing evolution of pain management strategies. Our study uniquely identifies the expanding application of the ERAS concept within MA, likely driven by the global emphasis on postoperative recovery and complication reduction. The prominence of journals like the Cureus Journal of Medical Science and Anesthesia and Analgesia in our findings mirrors their recognized role in disseminating pain management research. Influential authors such as Richard D. Urman and Henrik Kehlet have significantly contributed to the advancement of MA, as reflected in our analysis. Their work aligns with the broader academic community’s efforts to enhance pain management practices. However, our focused examination of MA research allows for a more precise assessment of their contributions within this specific domain.

By comparing our findings with previous bibliometric analyses, this study demonstrates its unique contributions and value in the field of multimodal analgesia. Additionally, the cited literature provides readers with resources for further exploration of this research area. Future studies could build on our findings to delve deeper into optimizing multimodal analgesia, fostering international collaborations, and exploring the application of emerging technologies in pain management.

### Limitation

This study provides a comprehensive analysis of MA research from 2013 to 2023 but has several limitations. Reliance on the WOSCC may introduce selection bias by excluding significant research from other databases, potentially leading to an incomplete representation of the field. Additionally, the 10-year time frame might miss recent trends or emerging studies not yet widely cited. Language bias is another concern, as the majority of analyzed publications are likely in English, excluding significant contributions from non-English-speaking countries, limiting the global perspective. The analysis identifies major contributing countries and institutions but lacks a detailed examination of the nature and impact of these collaborations. Furthermore, the study points out emerging research areas but does not comprehensively analyze how these areas are evolving and their potential future impact. Another limitation is the potential underestimation of multidisciplinary contributions, as it may not fully capture the interconnectedness of MA research with other fields such as pharmacology, nursing, and physical therapy, leading to a narrower understanding of collaborative efforts that enhance patient care.

## Conclusion

This bibliometric analysis provides an in-depth overview of MA research in the perioperative period from 2013 to 2023. By examining data from the WOSCC, the study identifies key trends, influential authors, leading institutions, and contributing countries. The USA, China, and Canada emerge as the primary contributors, reflecting a global focus on this topic. Richard D. Urman and Henrik Kehlet are recognized as prominent figures in the field. Key journals, such as the Cureus Journal of Medical Science and Anesthesia and Analgesia, play crucial roles in disseminating research. Research hotspots identified include “opioids,” “TKA,” and “ERAS.” The analysis underscores the significance of MA in improving postoperative outcomes and emphasizes the need for further research, particularly in optimizing rehabilitation parameters and enhancing patient recovery protocols.

Future research should focus on optimizing rehabilitation within MA protocols to better integrate pain management with recovery. Additionally, studying the long-term effects of opioid-sparing strategies on quality of life and evaluating new regional anesthesia techniques, such as ultrasound-guided nerve blocks, would offer valuable insights. Further exploration is needed on adapting MA protocols to diverse patient populations, including those with comorbidities, to ensure personalized care. Lastly, fostering international collaborations to assess the global applicability of MA strategies, especially in developing healthcare settings, will help create universal frameworks for perioperative pain management.

## Data Availability

The raw data supporting the conclusions of this article will be made available by the authors without undue reservation.

## Supplementary material

SUPPLEMENTARY FIGURE 1 Visualization of international cooperation between countries. Chord diagram illustrating the patterns of international cooperation between countries. Each sector in the diagram represents a country, and the color coding distinguishes different entities. The width of the chords is proportional to the strength of the cooperative relationship between the countries, with wider chords indicating stronger cooperation. The diagram highlights both bilateral and multilateral cooperation, visualizing the flow of interactions across nations.
