# Supplementary figures and images for: Bibliometric analysis of multimodal analgesia research in the perioperative period: trends, contributions, and emerging areas (2013–2023)
Source: Front Med (Lausanne). 2025 Apr 3;12:1573112. doi: 10.3389/fmed.2025.1573112 (PMC12004494; doi:10.3389/fmed.2025.1573112)

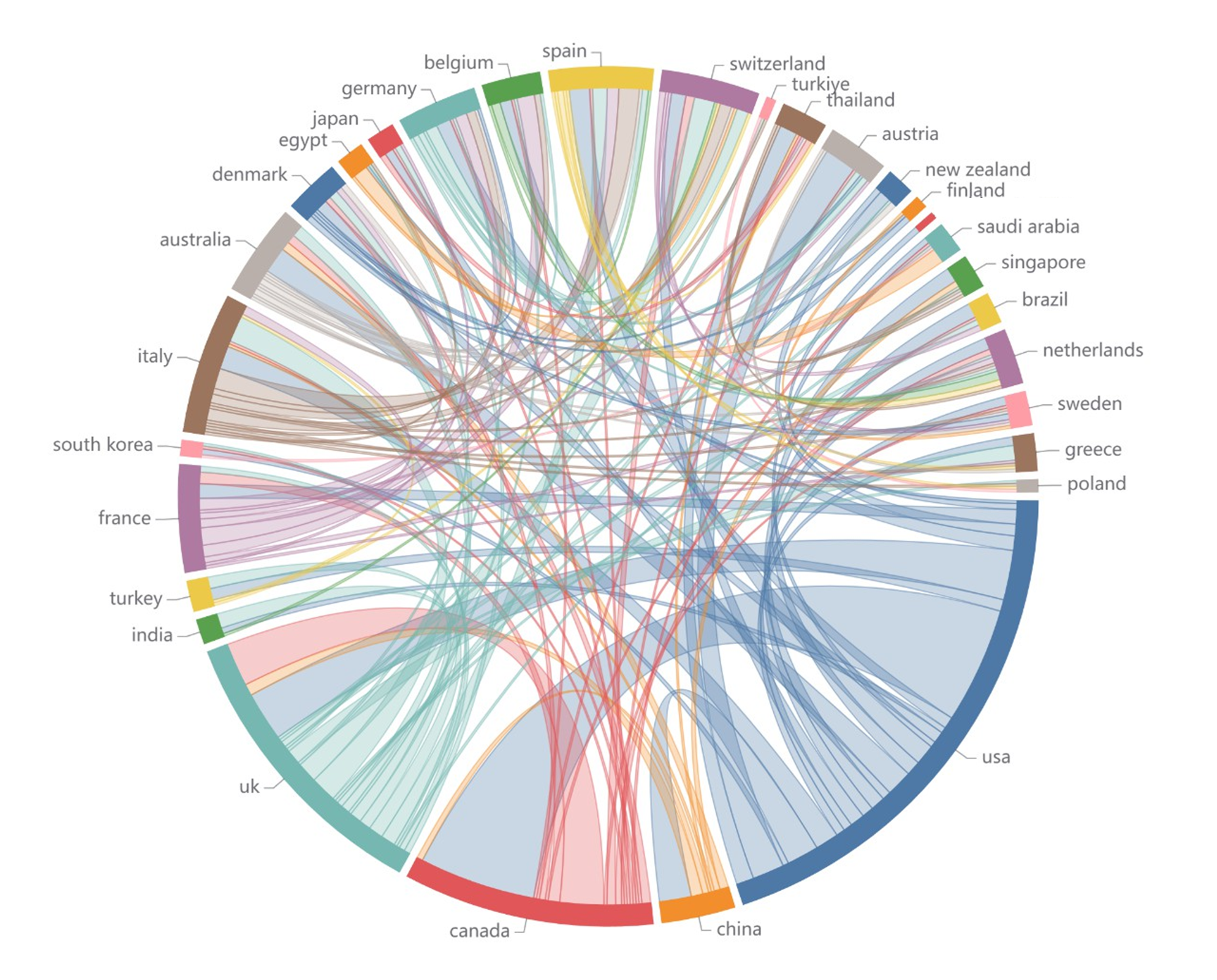

Supplement: SUPPLEMENTARY FIGURE 1 — Visualization of international cooperation between countries. Chord diagram illustrating the patterns of international cooperation between countries. Each sector in the diagram represents a country, and the color coding distinguishes different entities. The width of the chords is proportional to the strength of the cooperative relationship between the countries, with wider chords indicating stronger cooperation. The diagram highlights both bilateral and multilateral cooperation, visualizing the flow of interactions across nations. [file Image_1.tif]
